# An acetylcholinesterase biosensor with high stability and sensitivity based on silver nanowire–graphene–TiO_2_ for the detection of organophosphate pesticides†

**DOI:** 10.1039/c9ra02140j

**Published:** 2019-08-13

**Authors:** Jianhua Zhang, Bo Wang, Yiru Li, Wenhao Shu, Huaying Hu, Lianqiao Yang

**Affiliations:** Key Laboratory of Advanced Display and System Applications, Ministry of Education, Shanghai University Yanchang Road 149 Shanghai 200072 China yanglianqiao@i.shu.edu.cn

## Abstract

An electrochemical acetylcholinesterase biosensor based on silver nanowire, graphene, TiO_2_ sol–gel, chitosan and acetylcholinesterase has been fabricated successfully for the detection of organophosphate pesticides. The outstanding electrical properties of silver nanowires and graphene, and moreover the self-assembly of these two nanomaterials make the biosensor highly sensitive. Simultaneously, the immobilization efficiency of the enzyme is greatly improved by the action of the TiO_2_ fixed matrix. Under optimum conditions, the biosensor exhibited excellent performance for the detection of dichlorvos with a linearity in the range of 0.036 μM to 22.63 μM and the detection limit was found to be 7.4 nM. The biosensor was highly reproducible and stable during detection and storage.

## Abstract 2

An electrochemical acetylcholinesterase (AChE) biosensor based on silver nanowire, graphene stripped by 1-methyl-2-pyrrolidinone, TiO_2_ sol–gel, chitosan and AChE has been fabricated successfully for the detection of organophosphate pesticides.

## Graphical abstract

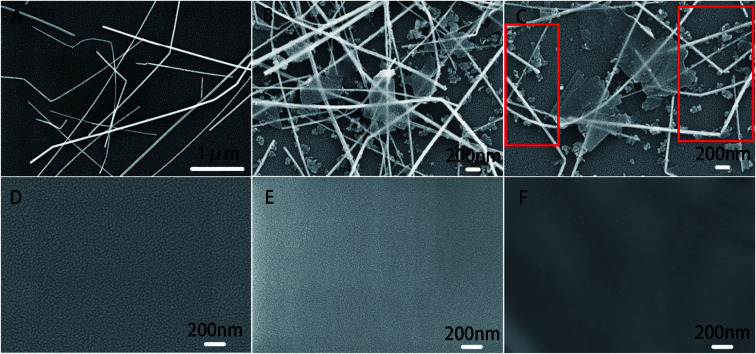

## 1. Introduction

Organophosphorus pesticides (OPs) have been widely used to protect crops from insects for decades. Its widespread utilization in agricultural production has resulted in varying degrees of residue in crops. However, OPs cause an accumulation of acetylcholine (ATCl) in humans by inhibiting the activity of acetylcholinesterase (AChE), excessively stimulating receptors in synapses and ultimately causing damage to the nervous system, and they are toxic to humans and most animals.^1–5^ It is very important to develop accurate, quick, easy and convenient detecting approaches for OP residues in agricultural products.

Traditional methods for detecting pesticide residues are liquid chromatography and mass spectrometry.^6,7^ Although these methods produce effective results, they are time consuming and require specialized technicians for analysis.^8^ In recent years, many other methods have been developed to detect OPs, including electrochemistry,^9,10^ fluorometry,^11,12^ colorimetry,^13^*et al.* Among these methods, electrochemistry method has attracted huge attention because of its rapid response, high sensitivity, and low-budget.^14–16^

Immobilization of AChE and signal magnification are everlasting issues and require consideration of both biocompatibility and conductivity for AchE biosensors. It is well known that viscous CS is a good immobilized polymer since it can immobilize the enzyme in a simple manner. Immobilization of acetylcholinesterase with TiO_2_–CS composite film has been approved to be a more effective approach by Cui *et al.*^17^ For the signal magnification, various low dimension materials have been tried and achieved certain improvements, including TiO_2_–CS,^17^ calcium carbonate–chitosan,^18^ Au NCs–CeO_2_ NWs,^19^ Co–2Ni–B,^20^ carbon nanotube^21^ and AuNPs–PPy nanowires,^22^ as shown in Table 1.^17–25^ Until now, silver nanowires (AgNWs) are rarely used in AchE biosensors even if it has excellent electrical properties and have been used in the field of transparent electrodes for optoelectronic devices,^26–30^ possibly due to its unstability to water and oxygen. In addition to the excellent opto-electronic performance, both Gra and AgNWs are biocompatible nanomaterials that can be used on biosensors as amplification strategies.

**Table 1:** Comparison with literature reported AChE biosensors

Electrode material	Linear range/μM	Detection limit/nM	Analyte	References
Gra/AgNWs	0.036–22.63	7.4	DDVP	This work
RGO	0.036–22.6	29	DDVP	Cui *et al.*^17^
Calcium carbonate–chitosan	0.018–0.759 and 2.84–14.24	3.7	Methyl parathion	Gong *et al.*^18^
Au NCs–CeO_2_ NWs	5.0 × 10^−4^–470	0.17	ATCl	Zhang *et al.*^19^
Co–2Ni–B	3 × 10^−6^–0.3	2.83 × 10^−3^	Chlorpyrifos	Lu *et al.*^20^
Carbon nanotube	Up to 200	50	Methyl parathion	Lin *et al.*^21^
AuNPs–PPy nanowires	0.018–0.45 and 1.89–17.0	7.5	Methyl parathion	Gong *et al.*^22^
Bromothymol blue doped sol–gel film	0.14–5.7	110	Chlorpyrifos	Kuswandi *et al.*^23^
RGO–cellulose microfibers	Up to 1123	8	Fenitrothion	Velusamy *et al.*^24^
CdSe@ZnS/Gra	10^−6^–1	10^−3^	DDVP	Li *et al.*^25^

**Scheme 1:**
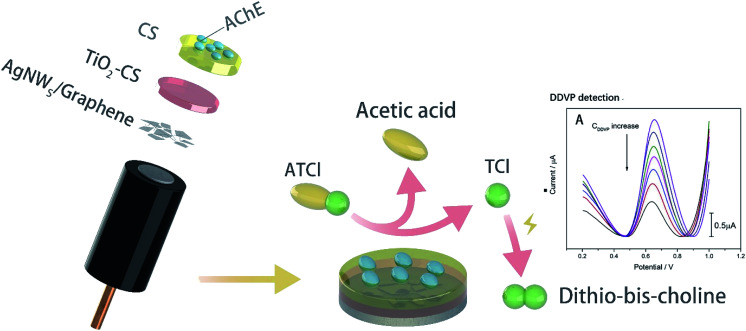
Schematic illustration of the AChE biosensor structure and its working mechanism to ATCl.

In this study, biosensor with AChE/CS/TiO_2_–CS/Gra/AgNWs structure (Scheme 1) has been constructed by adding AgNWs and Gra to the surface of glassy carbon (GC) electrode as a magnification strategy, using CS-doped TiO_2_ sol gel as an immobilization platform, and CS as an adhesive to fulfil effective adhesion to AChE. The sensitivity and stability of biosensor was evaluated. And storage experiment was carried out to test the catalytic activity durability. Finally, lettuce was use as the actual vegetable sample to verify its sensitivity and practicality.

## 2. Experimental

### 2.1 Materials and chemicals

AChE (from electric eel), ATCl, AgNWs and DDVP were from Sigma-Aldrich. CS (viscosity > 400 mPa s) and Gra (stripped by 1-methyl-2-pyrrolidinone (NMP)) were from Aladdin Bio-Chem Technology (Shanghai, China). Glucose was from Sangon Biotech (Shanghai, China), ethanol absolute, glacial acetic acid, tetra-*n*-butyl titanate, hydrochloric acid, *N*,*N*-dimethylformamide (DMF), isopropanol, potassium ferricyanide, potassium ferrocyanide trihydrate, potassium chloride, sodium chloride, sodium dihydrogen phosphate, disodium hydrogen phosphate were from Sinopharm Chemical Reagent Co., Ltd (Shanghai, China). All chemical reagents used are of analytical grade. Nitrogen with a purity of 99.9% comes from Air Liquide (China) Holding Co., Ltd. The deionized water (DI water) used in the whole experiment was obtained by ion exchange method. Vegetable juice is a supernatant obtained by washing, grinding, filtering, and centrifuging fresh lettuce purchased from a nearby vegetable market (ESI†).

### 2.2 Apparatus and measurement

Field emission scanning electron microscope (FESEM, Ultra 55 Zeiss, Germany) was used for scanning electron microscope (SEM) characterization of samples. The GC working electrode (3 mm), Ag/AgCl/KCl (3 M) reference electrode, platinum wire counter electrode and CHI660E electrochemical workstation used in the experiment were from Shanghai Chenhua Instrument (China). The modified GC electrode was characterized by cyclic voltammetry (CV) in 5 mM K_3_[Fe(CN)_6_] supported by 1 M KCl, and electrochemical impedance spectroscopy (EIS) techniques in 0.1 M KCl containing equimolar [Fe(CN)_6_]^3−/4−^ (10/10 mM) with AC frequency from 0.1 Hz to 100 kHz.

### 2.3 Preparation of graphene and CS solution

The Gra used in the experiment was obtained by diluting Gra stripped with NMP intercalation. Specifically, the NMP intercalated Gra solution (4 mg ml^−1^) was sonicated for 30 min, diluted with DMF to 0.4 mg ml^−1^, and finally ultrasonicated for 30 min, sealed and stored for use.

The CS solution was prepared by dissolving 1 g of CS powder in 100 ml 1% (vol%) glacial acetic acid to obtain a 1% CS solution, and then diluting the 1% CS solution to various concentrations with DI water.

### 2.4 Preparation of TiO_2_ sol–gel, and TiO_2_–CS mixture

The TiO_2_–CS sol–gel used to modify the GC electrode is a mixture of TiO_2_ precursor solution and 0.002% CS solution, which can be rapidly dried in air to form a film. The TiO_2_ sol–gel is mixed with tetra-*n*-butyl titanate, glacial acetic acid, DI water, and ethanol absolute in a specific molar ratio (tetra-*n*-butyl titanate : glacial acetic acid : DI water : and ethanol absolute = 1 : 1 : 4 : 50). First, the solution A was obtained by mixing 2.8 ml tetra-*n*-butyl titanate, 20.55 ml ethanol absolute, and 0.75 ml glacial acetic acid; the solution B was obtained by mixing 0.6 ml of water and 1.2 ml of ethanol absolute (adjust the pH to 1–2 with hydrochloric acid). The solution A was stirred for 30 min, then the solution B was added dropwise, and finally stirred for 30 min. The obtained TiO_2_ sol–gel was placed in an environment of 4 °C and sealed for use.

### 2.5 Fabrication of the biosensor

The GC electrodes were polished to a mirror surface with 0.3 and 0.05 mm alumina powder consecutively, then ultrasonically washed with DI water and absolute ethanol, and finally dried with high purity nitrogen. AgNWs was diluted to 2 mg ml^−1^ with isopropanol, and then 4 μl of AgNWs was added dropwise to the surface of the GC electrode, and dried under vacuum. Then 4 μl of Gra solution was added dropwise to the surface of the electrode, followed by vacuum drying. The TiO_2_ sol gel was dispersed in a CS solution (volume fraction of 0.002%) by a volume ratio of 99 : 1 to obtain a TiO_2_–CS mixed solution. 4 μl of TiO_2_–CS sol gel was added dropwise to the surface of the electrode, dried in air, and placed in a 0.2% CS solution for 20 s at a voltage of −2.5 V. After coating of CS to the electrode and drying in air, 4 μl of AChE diluted to 5 mg ml^−1^ with 1% bovine serum albumin (BSA) solution was added dropwise. And then the fabricated biosensor was placed in a 4 °C environment and stored for use.

## 3. Results and discussions

**Fig. 1:**
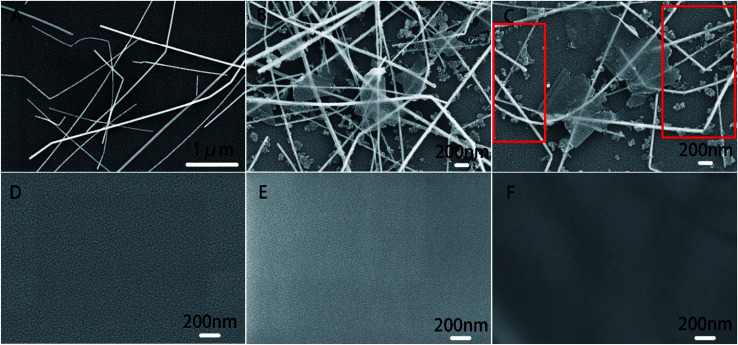
SEM images of (A) AgNWs/SiO_2_, (B) and (C) Gra/AgNWs/SiO_2_, (D) TiO_2_–CS/Gra/AgNWs/SiO_2_, (E) CS/TiO_2_–CS/Gra/AgNWs/SiO_2_ and (F) AChE/CS/TiO_2_–CS/Gra/AgNWs/SiO_2_ (second electron detector). The concentration of AgNWs is 2 mg ml^−1^ and AChE is 5 mg ml^−1^.

The structure of biosensor as-produced is shown in Scheme 1. The surface topography of each layer of the prepared electrode was characterized by SEM and the results were shown in Fig. 1. All samples were dispensed onto silicon wafers. The AgNWs (Fig. 1(A)) uniformly overlapped the surface of the silicon wafer to form a conductive mesh. Gra (Fig. 1(B)) intercalated by NMP covered the surface of AgNWs, enhancing the connection at the node. In addition to large-scale Gra enhancing the connection of AgNWs network nodes in a cover-up manner, small pieces of Gra (red rectangle area in Fig. 1(C)) self-assembled around AgNWs, enhancing the coverage area of the AgNWs network. Subsequently, TiO_2_–CS was dropped on the surface of the AgNWs and Gra nano-film to uniformly and evenly cover the surface of the silicon wafer. Uniform nano-particle protrusions with diameter of ∼23 nm were formed on the surface (Fig. 1(D)). After the CS is coated to the surface (Fig. 1(E)) of the silicon wafer, the surface morphology becomes finer, more uniform, and the surface nanoparticles have a diameter of ∼15 nm. The AChE (Fig. 1(F)), which was finally immobilized on the surface of the CS, makes the entire flat smoother and slightly undulating.

**Fig. 2:**
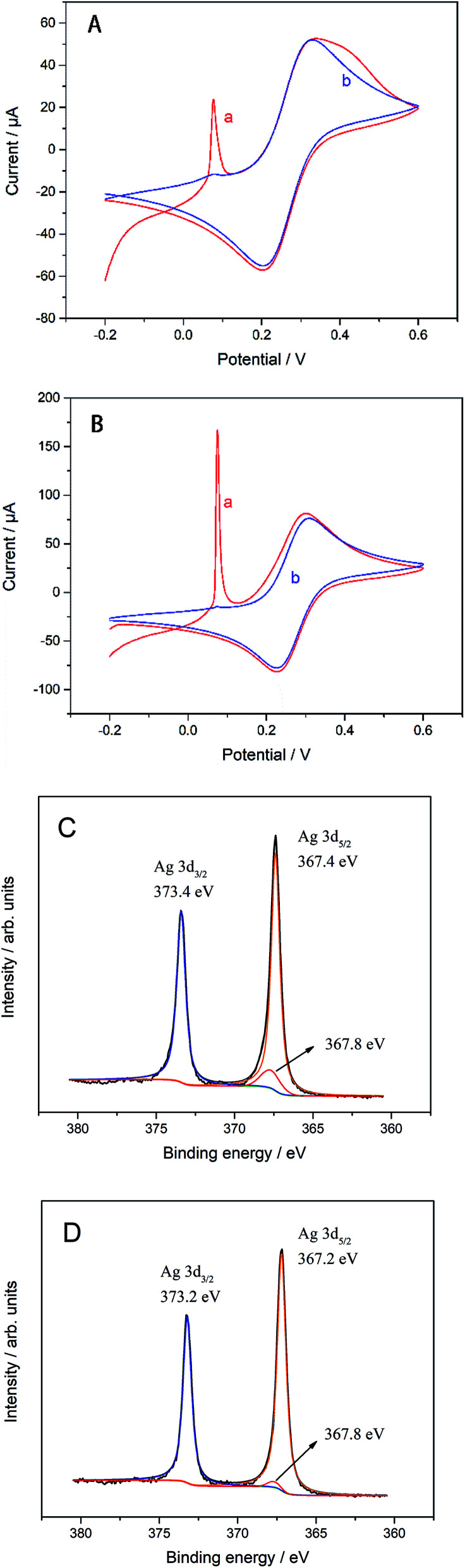
CV of (A) AgNWs/GC and (B) Gra/AgNWs/GC electrode, where (a) cycle 1^st^ and (b) cycle 15^th^, and XPS spectra of Ag 3d in (C) AgNWs/SiO_2_ and (D) Gra/AgNWs/SiO_2_ samples after annealed in atmosphere at 120 °C for 12 h.

The electrode preparation process was also characterized by electrochemical techniques. The CV of GC electrode with or without modification in K_3_[Fe(CN)_6_] and DPV of biosensors with or without modified by AgNWs is shown in Fig. 2(A) and (B). It can be seen clearly that there is a sharp oxidation peak around 0.1 V, and gradually weakens to almost invisible after 15 cycles in Fig. 2(A) and (B). Compared with the curve a in Fig. S1(A),† the CV performance of bare GC electrode, which is no obvious peak at 0.1 V. Thus, the peak at about 0.1 V can be considered to be caused by irreversible oxidation of AgNWs at a forward voltage, so that the peak current decreased to be almost invisible after several cycles. The more obvious oxidation peak at 0.1 V for the electrode modified by Gra and AgNWs should be due to the signal amplification effect of graphene to the oxidation current of AgNWs. In Fig. 2(A), the oxidation peak due to [Fe(CN)_6_]^−^ oxidation was not improved at around 0.29 V with the modification of AgNWs on GC electrode. However, after Gra is covered on the surface of the GC electrode in Fig. 2(B), the peak current of about 0.29 V was also significantly improved after several cycles and higher than the one which was modified by Gra only. In Fig. S1(A),† we can clearly see the CV curve of different modified electrodes. Briefly, the oxidation peak of curve c which was modified by AgNWs and Gra was higher than any other one. In particular, it was higher than the oxidation peak of the electrode with only Gra modified. Considering that Gra can protect AgNWs from oxidation,^31^ so that we can believe that due to the coverage protection of GC, AgNWs were not fully oxidized, and the synergistic effect of the remaining AgNWs and Gra improves the electrical properties of the GC electrode. And the XPS spectra in Fig. 2(C) and (D) can prove incomplete oxidation of AgNWs. As shown in Fig. 2(C), AgNWs in AgNWs/SiO_2_ still retains its intrinsic metal state (Ag 3d_5/2_: 367.4 eV), while also exhibiting a distinct Ag_2_O (Ag 3d_5/2_: 367.8 eV) oxidation state, while the satellite peak of AgNWs in Fig. 2(D) is small. Accordingly, unlike Gra/AgNWs/SiO_2_, AgNWs/SiO_2_ exhibits Ag_2_O oxidation (O 1s: 530.9 eV) states in its O 1s spectra in Fig. S1(C) and (D),† respectively.

As comparison, biosensors only modified by Gra was also fabricated and the results are shown in Fig. S1(B).† The DPV curve of the biosensor has a significant oxidation peak around 0.62 V due to oxidation of the thiocholine (TCl) generated by ATCl decomposition. It can be clearly seen that the biosensor modified by Gra and AgNWs has a higher oxidation peak, which proves that the addition of AgNWs can further enhance the amplification effect.

**Fig. 3:**
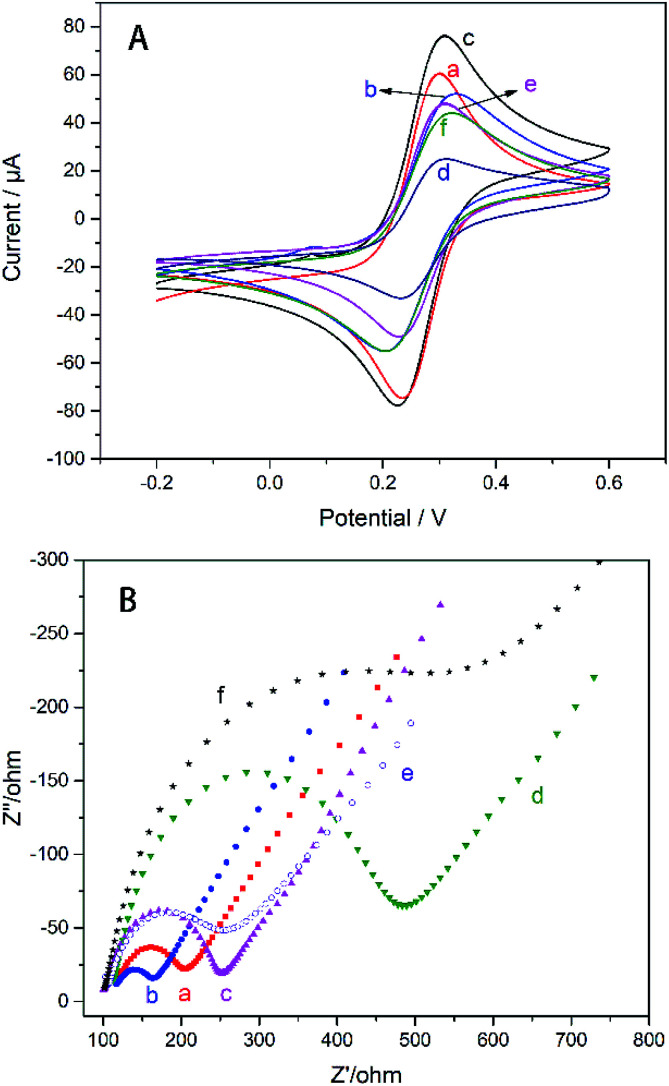
(A) The CV and (B) the EIS of (a) GC, (b) AgNWs/GC, (c) Gra/AgNWs/GC, (d) TiO_2_–CS/Gra/AgNWs/GC, (e) CS/TiO_2_–CS/Gra/AgNWs/GC, (f) AChE/CS/TiO_2_–CS/Gra/AgNWs/GC electrodes, [Fe(CN)_6_]^3−/4−^ (10/10 mM). AChE concentration: 5 mg ml^−1^.

The electrochemical properties of the modified electrodes were probed with K_3_[Fe(CN)_6_]. From Fig. 3 the electrode modified only by AgNWs has a lower peak current (curve b) because the nano-silver on the electrode surface is oxidized and hinders the transport of electrons after multiple cycles. It can be seen from the Fig. 3 that the current curves of the upper and lower layers of the glassy carbon electrode modified with AgNWs and Gra are significantly increased (curve c). AgNWs and Gra hybrid nanomesh films significantly enhanced the conductivity of the GC electrode. According to eqn (1), effective area of the electrode can be calculated.^32^

1 *I*_p_ = 2.69 × 10^5^*n*^3/2^*AD*_0_^1/2^*C*_0_*ν*^1/2^

where *I*_p_, *n*, *A*, *D*_0_, *C*_0_ and *ν* represent the redox peak current (amperes), the electrons oxidized or reduced per molecule, the effective surface area of the electrode (cm^2^), and the diffusion coefficient of K_3_[Fe(CN)_6_] in 1 M KCl (0.76 × 10^−5^ cm^2^ s^−1^), concentration of redox species (mol cm^−3^) and scanning rate (V s^−1^). Calculation basing on eqn (1) shows that addition of AgNWs and Gra increase the effective area of the electrode from 0.071 to 0.089 cm^2^ (Fig. S2†). The introduction of TiO_2_–CS nanocomposites significantly reduced the redox current (curve d), due to the semi-conductivity of TiO_2_, the non-conductivity of CS and the negatively charged surface of TiO_2_ gel.

In contrast, the CS layer electrodeposited on the surface of TiO_2_–CS increased the redox current (curve e). Although the conductivity of CS is very poor, the positive charge on the surface of CS can effectively increase the adsorption of negative ions of K_3_[Fe(CN)_6_], and then the redox current is improved.^17^ The cover of AChE lead to the reduction of peak current due to the non-conductivity of AChE.

**Fig. 4:**
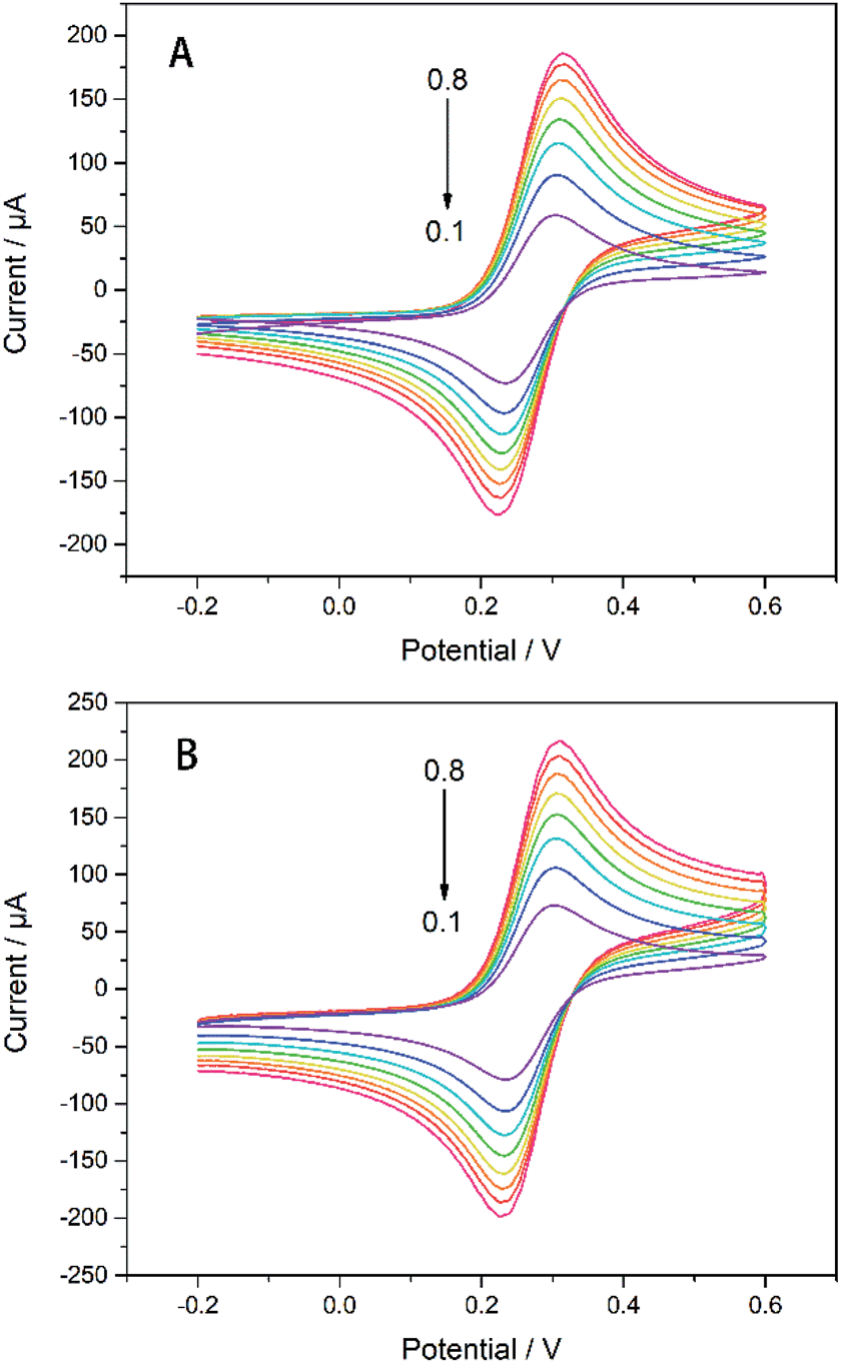
CVs of (A) bare GC electrode and (B) Gra/AgNWs/GC electrode in 5 mM K_3_[Fe(CN)_6_]/1 M KCl with different scan rate (from 0.1–0.8 V s^−1^).

It can be seen from Fig. 4 that as the scanning rate increases, the redox current is significantly improved, whether with or without modification by AgNWs and Gra. Both the current of the electrode modified by AgNWs and Gra at each scan rate are higher compared to the bare electrode. Moreover, the redox peak current (*I*_p_) has a good linear relationship with the square root of the sweep rate (Fig. S2†).

EIS technology is used to detect the membrane capacitance and resistance of the electrode and can also be used to characterize the sensor preparation process. The Nyquist complex plan of all electrodes presents a single semicircle in the high frequency domain, the diameter of which is related to the transmission impedance *R*_CT_, and the straight line in the low frequency domain is related to the diffusion process. The *R*_CT_ can be calculated from the diameter of the half cycle portion by eqn (2).^33^2
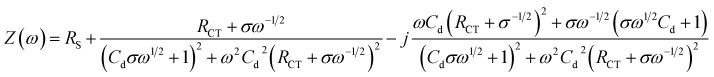
where *R*_S_ is the solution resistance, *C*_d_ is the double layer capacitance, *ω* = 2π*f*, and *f* is the frequency, 
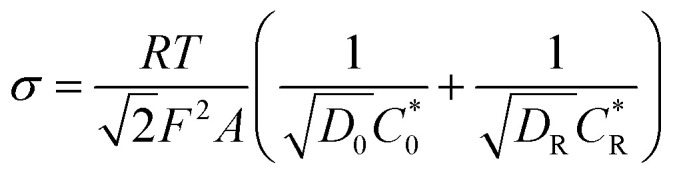
, where *R* is the gas constant, *T* is the absolute temperature, *F* is the Faraday constant, *A* is the electrode area, *D*_o_, *D*_R_ is the diffusion coefficient of the oxidant and reducing agent, and 
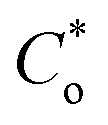
 and 
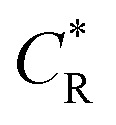
 are the oxidant and reducing agent concentration.

According to calculation, the *R*_CT_ of the GC electrode modified with AgNWs (unoxidized) is ∼39.6 Ω (curve b) lower than the ∼69.81 Ω (curve a) of the bare electrode. Therefore, unoxidized AgNWs have a positive effect on reducing the transmission impedance of the electrode. Thereafter, the *R*_CT_ of the electrode modified with Gra (curve c) is increased to ∼123.8 Ω because the effective area of the electrode is improved after the Gra modification. Subsequent addition of TiO_2_–CS nanofilm (curve d), CS film (curve e), AChE layer (curve f), lead to the variation of *R*_CT_ to ∼291.1, ∼94.92, and ∼320.2 Ω, respectively. Similar to CV, the semiconductor properties of TiO_2_ and the non-conductivity of CS lead to an increase in *R*_CT_; the positive charge accumulation of electroplated CS increases the charge transfer efficiency and reduces the impedance;^17^ finally, the addition of AChE dramatically increased *R*_CT_ due to its non-conductivity.

**Fig. 5:**
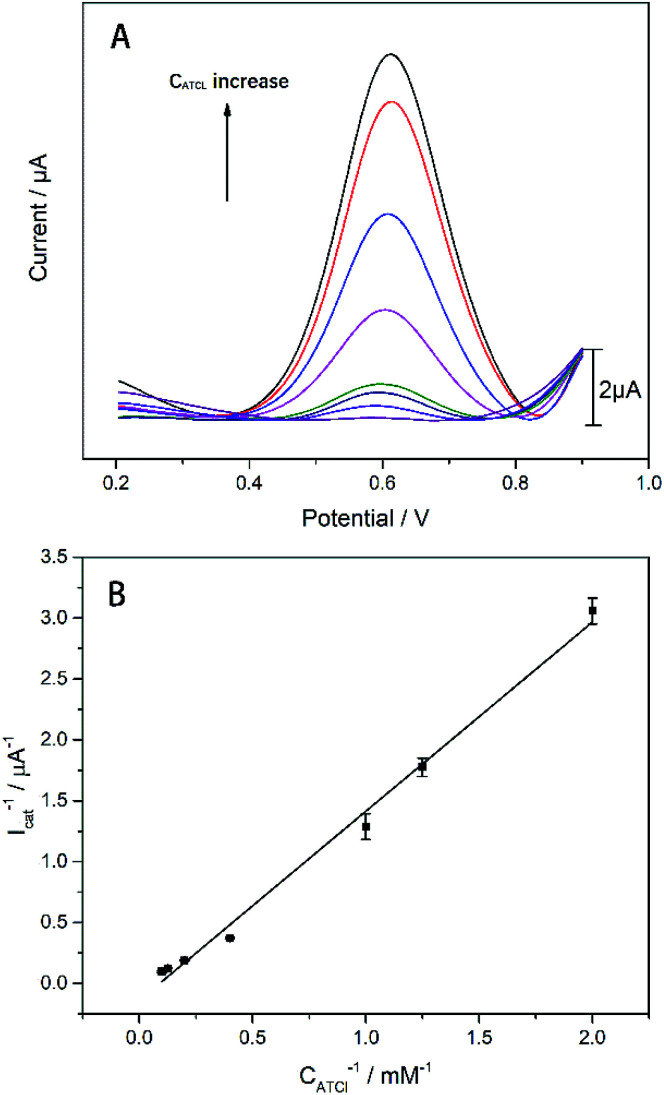
(A) The DPV responses of the AChE/CS/TiO_2_–CS/Gra/AgNWs/GC electrode to various concentrations of ATCl. (B) The plot of 1/*I*_cat_*versus* 1/*C*_ATCl_. *n* = 3.

The electrochemical activity of the biosensor is revealed by means of differential pulse voltammetry (DPV). The detection voltage of DPV ranges from 0.2 to 0.9 V, the amplitude is 0.05 V, the pulse width is 0.005 s, pulse period is 0.02 s, the concentration of ATCl is 0.2–9 mM, and it is dissolved in phosphate buffer saline (PBS) (pH 7.4). AChE can catalyze the decomposition of ATCl to produce thiocholine (TCl) and acetic acid, while the irreversible oxidation of TCl produces a distinct oxidation peak on the DPV curve (Scheme 1). The experimental results also demonstrate that an apparent DPV peak can be detected on an electrode with AChE compared to electrode without enzyme (Fig. S3†). It can be seen from Fig. 5(A) that as the concentration of ATCl increases, the oxidation peak current of the biosensor is significantly improved. The reciprocal of the peak current, *I*_cat_^−1^, is linear with the reciprocal *C*_ATCl_^−1^ of the concentration of ATCl: *I*_cat_^−1^ = 0.3745*C*_ATCl_^−1^ + 0.0807 (*R*^2^ = 0.9979). By using the Lineweaver–Burk equation (eqn (3)),^34^ the apparent Michaelis–Menten constant (*K*_m_) was obtained to be 4.6383 mM.3
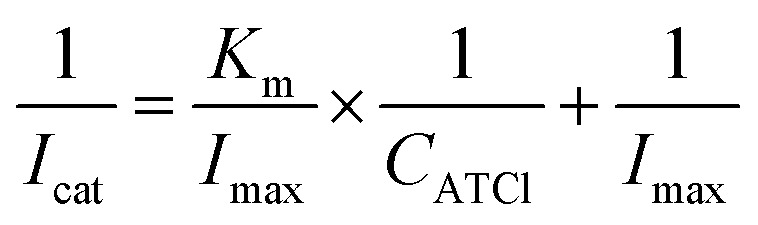
where *I*_max_ was the maximum current measured under saturated substrate condition.

**Fig. 6:**
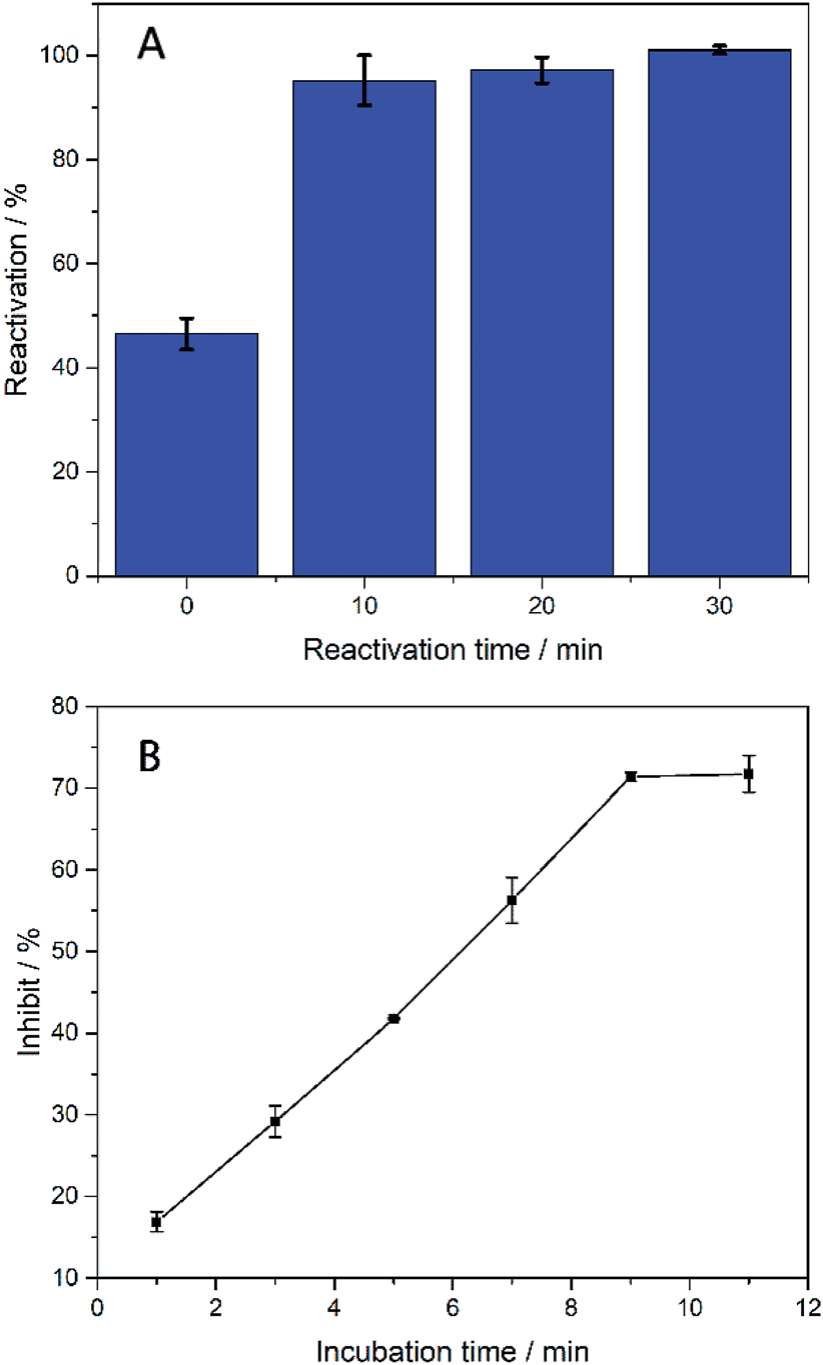
(A) Histogram of biosensor reactivation% of DDVP after incubation, *n* = 3–5; (B) inhibit% of biosensor with incubation time in DDVP. The concentration of DDVP is 22.63 μM. *n* = 3–5.

In order to make biosensors perform better, some optimization have been made to the parameters of the biosensor's testing process. In this study, dichlorvos (DDVP) was used as a representative of OPs. The biosensor can be reactivated in PBS while it inhibited by DDVP. The principle of AChE reactivation by PBS is not understood at this moment and corresponding mechanism needs further investigation.^35–38^ For each test accuracy, the biosensor was allowed to rest in the PBS environment for a period of time after undergoing OPs inhibition until the catalytic performance is fully reactivated. After the biosensor was inhibited by immersion in DDVP, it can be reactivated by being soaked in PBS. From Fig. 6(A), it can be clearly seen that the reactivation% at 0 min soaking in PBS was less than 50% (reactivation% = *I*_latter_/*I*_former_, where *I*_former_ and *I*_latter_ are the DPV peak currents in 1 mM ATCl for sensors that are not processed and processed by DDVP, respectively). After 10 min rest in PBS, reactivation% of the biosensor has been significantly improved, close to 100%, but its catalytic performance has not fully reactivated. After 30 min, the catalytic performance was fully restored, so 30 min was selected as the reactivation time. From Fig. 6(B) we can see significantly that the inhibit% (
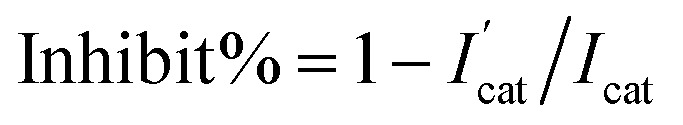
, where 
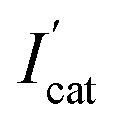
 and *I*_cat_ are the DPV peak currents in 1 mM ATCl for sensors that are processed and not processed by DDVP, respectively) of the biosensor increased with increasing incubation time (before 10 min) in the DDVP solution. While the incubation time increased to 9 and 11 min, the inhibit% of the biosensor did not change any more, so 10 min was selected as the incubation time of the sensor in DDVP.

**Fig. 7:**
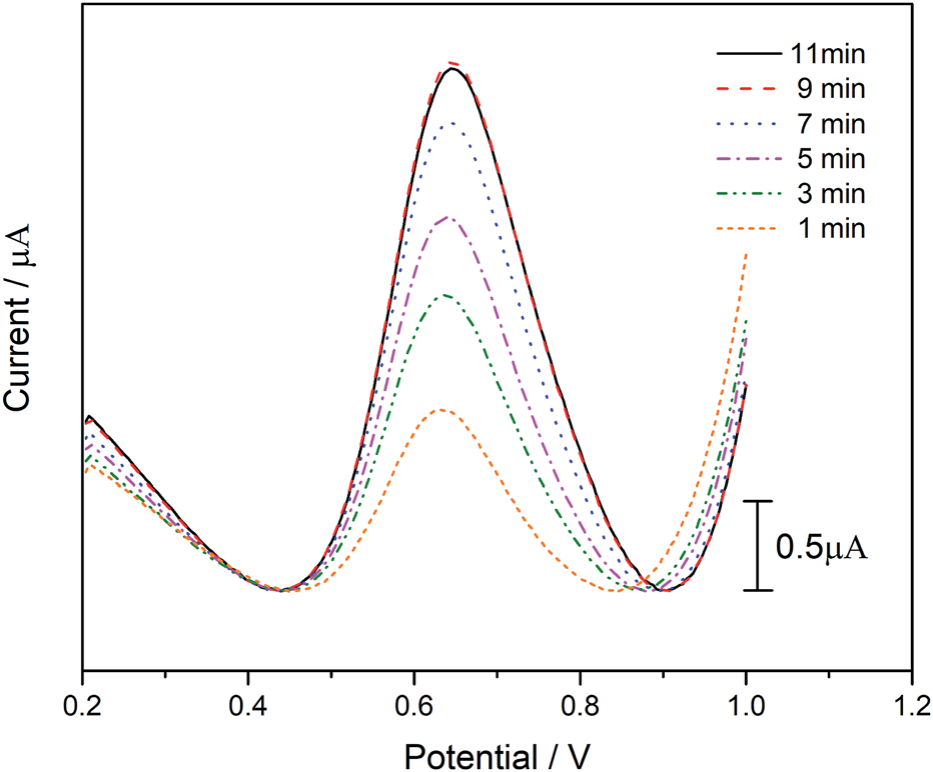
The DPV performance of the biosensor at different incubation times in 1 mM ATCl.

Due to the catalytic decomposition of ATCl is not instantaneous, we need to determine when it can perform most of the substrate decomposition. From Fig. 7, it can be seen clearly that the peak current of DPV increase with the incubation time at first, but no obvious change can be found when the incubation time increased from 9 to 11 min. To ensure that the biosensor prepared is capable of catalyzing most of the substrate, we set the catalytic incubation time to 10 min.

**Fig. 8:**
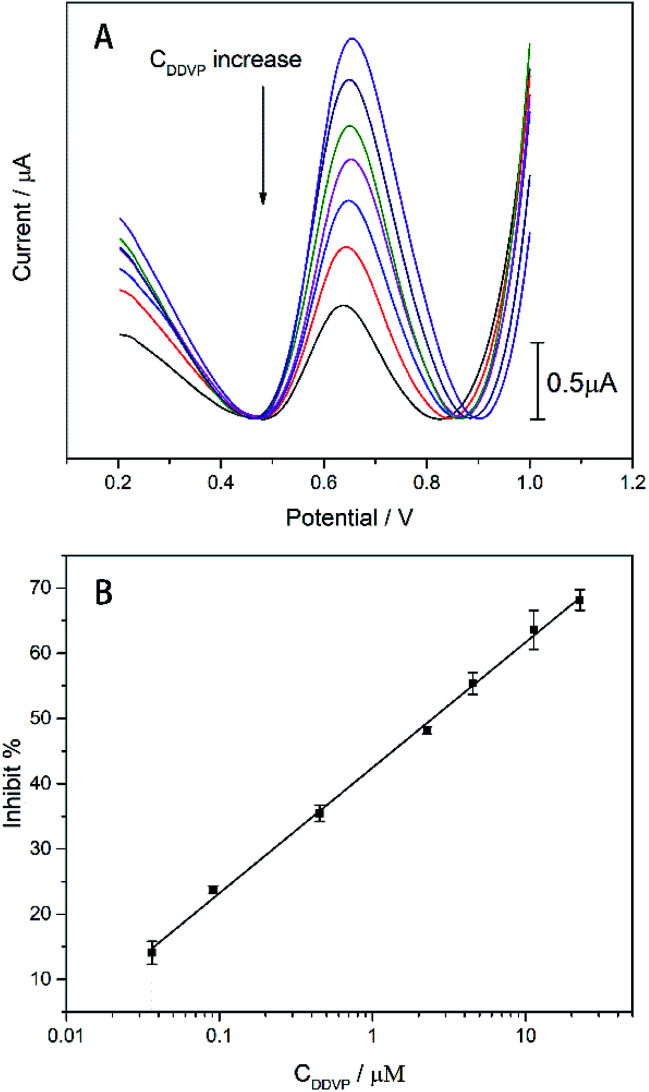
(A) The DPVs of the AChE/CS/TiO_2_–CS/G/AgNWs/GC electrode in 1 mM ATCl after incubated in different concentrations of DDVP. (B) The inhibit% values by various concentrations of DDVP. The concentration gradient of DDVP is: 0.036, 0.091, 0.45, 2.26, 4.53, 11.31 and 22.63 μM. The AChE concentration: 5 mg ml^−1^. *n* = 3–7.

The analysis of the DDVP by the enzyme biosensor can be revealed from Fig. 8. The DDVP test is carried out in the 1 mM ATCl standard solution. As shown in Fig. 8(A), the oxidation current generated by thiocholine (TCl) oxidation decreases with the concentration of DDVP due to the DDVP inhibition. The peak current dropped from 2.51 to 0.75 μA when the concentration of DDVP increased from 0.036 to 22.63 μM. We can calculate the inhibition rate of the enzyme biosensor under different concentrations of DDVP, and obtain the linear relationship as shown in Fig. 8(B). Least squares method was used to fit the linear relationship between inhibition rate and DDVP concentration: inhibit% = 19.2348 lg *C*_DDVP_ + 42.4512 (*R*^2^ = 0.9978).

The limit of detection (LOD) was 7.4 nM (1.64 ppb) (calculated in a 3*σ* rule). The LOD and minimum values in the linear range are lower than the EU Pesticide Database and the US reported DDVP (10 ppb) maximum limits (MRLs). Besides that, the LOD and linear range of the biosensor are larger than most reported values. In addition, it is worth noting that our biosensor's lowest value in the linear range (1.64 ppb) is superior to most reported values (Table 1).

**Fig. 9:**
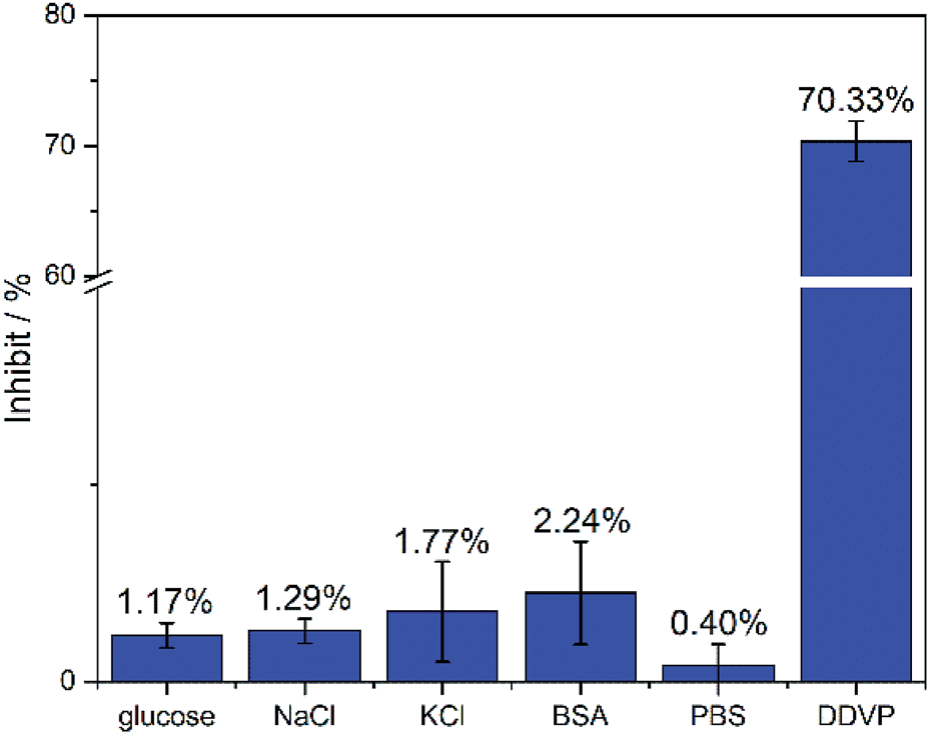
Inhibit% of biosensors under different analytes. The concentration of glucose, NaCl, KCl, and DDVP is 22.63 μM, and the concentration of BSA is 1 mg ml^−1^. *n* = 3–5.

In particular, the fabricated biosensors are extremely stable, adaptable and has well selectivity in glucose, NaCl, KCl, BSA, PBS and DDVP as showed in Fig. 9. The inhibit% of DDVP on the manufactured biosensor is 70.33%, which is much higher than the inhibit% of glucose, NaCl, KCl, BSA and PBS (less than 3%). The biosensor can be immersed in PBS for one month at room temperature without obvious changes in catalytic performance (Fig. S4†). Simultaneously, three identical biosensors were fabricated with relative standard deviation (RSD%) of only 2.128%, indicating that the biosensor is reproducible (Fig. S5†). In addition, the biosensors are also capable of detecting actual samples. The biosensor accurately distinguishes the one that is doped with DDVP in vegetable juice samples. The performance of biosensor in vegetable juice samples was comparable to that in PBS solution with DDVP (Table 2), which indicates the strong anti-interference even in complex solutions such as vegetable juice.

**Table 2:** The DDVP recovery ratios in lettuce juice samples detected with the biosensor

Sample no.	DDVP added (μM)	DDVP detected (μM)	Recovery (%)	RSD (%) (*n* = 3)
1	0.036	0.033	92.7	10.218
2	0.453	0.432	95.5	2.873
3	11.313	12.553	110.9	3.805

## 4. Conclusions

In this paper, a highly sensitive and stable multilayer structure AChE biosensor: AChE/CS/TiO_2_–CS/Gra/AgNWs/GC electrode, is fabricated by layer-by-layer stacking. Especially, the amplification strategy of the biosensor is fulfilled by self-assembly of AgNWs and Gra. A linear relationship between inhibit% and logarithm of DDVP concentration in the range of 0.036 to 22.63 μM was obtained and the LOD is 7.4 nM. Selectivity of the biosensors have also been verified by comparing the inhibit% with other analytes and utilization in real vegetable sample. In addition, the biosensors can be stored under mild conditions, just immersed in PBS, even low temperature conditions are not necessary. The excellent performance of biosensor makes it a good reference to related researchers and a promising candidate in the detection of organophosphate pesticides.

## Conflicts of interest

There are no conflicts to declare.

## Supplementary Material

RA-009-C9RA02140J-s001

## References

[cit1] Jokanovićab M., Kosanović M. (2010). Environ. Toxicol. Pharmacol..

[cit2] Costa L. G. (2006). Clin. Chim. Acta.

[cit3] Eddleston M., Buckley N. A., Eyer P., Dawson A. H. (2008). Lancet.

[cit4] Patočka J., Kuča K., Jun D. (2004). Acta Med..

[cit5] Long Q., Li H., Zhang Y., Yao S. (2015). Biosens. Bioelectron..

[cit6] Radišić M., Grujić S., Vasiljević T., Laušević M. (2009). Food Chem..

[cit7] Huang G., Ouyang J., Baeyens W. R. G., Yang Y., Tao C. (2002). Anal. Chim. Acta.

[cit8] Zhai C., Sun X., Zhao W., Gong Z., Wang X. (2013). Biosens. Bioelectron..

[cit9] Si Y., Zhang N., Sun Z., Li S., Zhao L., Li R., Wang H. (2014). Analyst.

[cit10] Hassani S., Akmal M. R., Salek-Maghsoudi A., Rahmani S., Ganjali M. R., Norouzi P., Abdollahi M. (2018). Biosens. Bioelectron..

[cit11] Meng X., Wei J., Ren X., Ren J., Tang F. (2013). Biosens. Bioelectron..

[cit12] Hou J., Tian Z., Xie H., Tian Q., Ai S. (2016). Sens. Actuators, B.

[cit13] Pohanka M., Karasova J. Z., Kuca K., Pikula J., Holas O., Korabecny J., Cabal J. (2010). Talanta.

[cit14] Li Y., Bai Y., Han G., Li M. (2013). Sens. Actuators, B.

[cit15] Zhou Q., Yang L., Wang G., Yang Y. (2013). Biosens. Bioelectron..

[cit16] Xu J., Yu C., Feng T., Liu M., Li F., Wang Y., Xu J. (2018). Nanoscale.

[cit17] Cui H. F., Wu W. W., Li M. M., Song X., Lv Y., Zhang T. T. (2018). Biosens. Bioelectron..

[cit18] Gong J., Liu T., Song D., Zhang X., Zhang L. (2009). Electrochem. Commun..

[cit19] Zhang C., Fan Y., Zhang H., Chen S., Yuan R. (2019). Anal. Bioanal. Chem..

[cit20] Lu X., Li Y., Tao L., Song D., Wang Y., Li Y., Gao F. (2018). Nanotechnology.

[cit21] Lin Y., Lu F., Wang J. (2004). Electroanalysis.

[cit22] Gong J., Wang L., Zhang L. (2009). Biosens. Bioelectron..

[cit23] Kuswandi B., Fikriyah C., Gani A. (2008). Talanta.

[cit24] Velusamy V., Palanisamy S., Chen S., Balu S., Yang T., Banks C. (2019). Talanta.

[cit25] Li X., Zheng Z., Liu X., Zhao S., Liu S. (2015). Biosens. Bioelectron..

[cit26] Geim A. K., Novoselov K. S. (2007). Nat. Mater..

[cit27] Xu Y., Liu J. (2016). Small.

[cit28] Han K., Xie M., Zhang L., Yan L., Wei J., Ji G., Luo Q., Lin J., Hao Y., Ma C. (2018). Sol. Energy Mater. Sol. Cells.

[cit29] Deng B., Hsu P. C., Chen G., Chandrashekar B. N., Liao L., Ayitimuda Z., Wu J., Guo Y., Lin L., Zhou Y., Aisijiang M., Xie Q., Cui Y., Liu Z., Peng H. (2015). Nano Lett..

[cit30] Kim H., Park Y., Choi D., Chu W. S., Ahn S. H., Chun D. M., Lee C. S. (2018). Appl. Surf. Sci..

[cit31] Kim S. H., Choi W. I., Kim K. H., Yang D. J., Heo S., Yun D. J. (2016). Sci. Rep..

[cit32] Hamann C. H. , Hamnett A. and Vielstich W., Electrochemistry, Wiley/VCH, Weinheim, Germany, 1998

[cit33] Bard A. J. and Faulkner L. R., Electrochemical Methods, John Wiley and Sons, New York, 2001

[cit34] Kamin R. A., Wilson G. S. (1980). Anal. Chem..

[cit35] Taylor P., Yan-Jye S., Momper J., Hou W., Andrea Camacho-Hernandez G., Radic' Z., Rosenberg Y., Kovarik Z., Sit R., Sharplesse K. B. (2019). Chem.-Biol. Interact..

[cit36] Shamagsumova R. V., Shurpik D. N., Padnya P. L., Stoikov I. I., Evtugyna G. A. (2015). Talanta.

[cit37] Li Y., Shi L., Han G., Xiao Y., Zhou W. (2017). Sens. Actuators, B.

[cit38] Li Y. P., Zhao R. X., Han G. Y., Xiao Y. M. (2018). Electroanalysis.

