# Supplementary material for: An acetylcholinesterase biosensor with high stability and sensitivity based on silver nanowire–graphene–TiO2 for the detection of organophosphate pesticides
Source: RSC Adv. 2019 Aug 13;9(43):25248–56. doi: 10.1039/c9ra02140j (PMC9069864; doi:10.1039/c9ra02140j)
Supplement: RA-009-C9RA02140J-s001 [file RA-009-C9RA02140J-s001.pdf]

Electronic Supplementary Material (ESI) for RSC Advances.
This journal is © The Royal Society of Chemistry 2019

An acetylcholinesterase biosensor with high stability and sensitivity based on silver nanowire-graphene-TiO_2 for the detection of organophosphate pesticides

Jianhua Zhang, Bo Wang, Yiru Li, Wenhao Shu, Huaying Hu, and Lianqiao Yang*

Key Laboratory of Advanced Display and System Applications, Ministry of Education, Shanghai University, Yanchang Road 149, Shanghai 200072, China

E-mail: yanglianqiao@i.shu.edu.cn

1. Experimental

Electrocatalytic and sensing performance

The electrocatalytic performance of ATCl was revealed by DPV technology. First of all, the prepared biosensor was immersed in PBS (pH 7.4) solution. Afterwards, it was circulated several times using CV technology until the curve was stable, so that the weakly adhered AChE was washed away. Thereafter, 5 ml, 1mM of ATCl was catalyzed at room temperature (25°C) for 10 minutes and characterized by DPV technique.

Processing of DDVP gradient solution and vegetable juice

The DDVP was diluted to a standard solution of 1 mg/mL with a methanol solution. Then it was diluted to 0.036, 0.091, 0.453, 2.263, 4.525, 11.313 and 22.627 μM with PBS.

The fresh vegetables purchased from the nearby market were washed, chopped, ground, and extruded to obtain the initial vegetable juice. The initial vegetable juice was filtered and placed in a centrifuge for centrifugation at 5000 rpm for 15 minutes. Then the supernatant was taken and diluted ten times with PBS for later use. Final, it was used to dilute DDVP standard to the specified concentration.

Storage experiment

The biosensor produced was tested by CV several times in PBS until its curve was stable, and then it was tested multiple times with DPV in 1 mM ATCl. Afterwards, it was immersed in PBS, stored at room temperature and taken out every few days to check its performance.

2. Results and discussion

2.1. Electrodes with or without Gra cover protection.

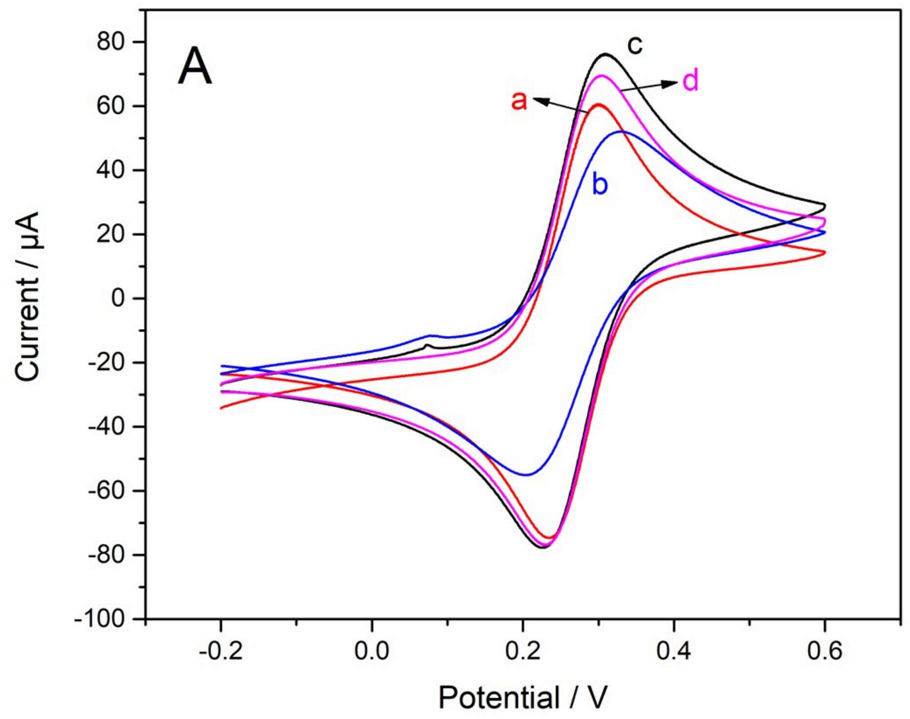
A
a
b
c
d
Current / μA
Potential / V
80
60
40
20
0
-20
-40
-60
-80
-100
-0.2
0.0
0.2
0.4
0.6

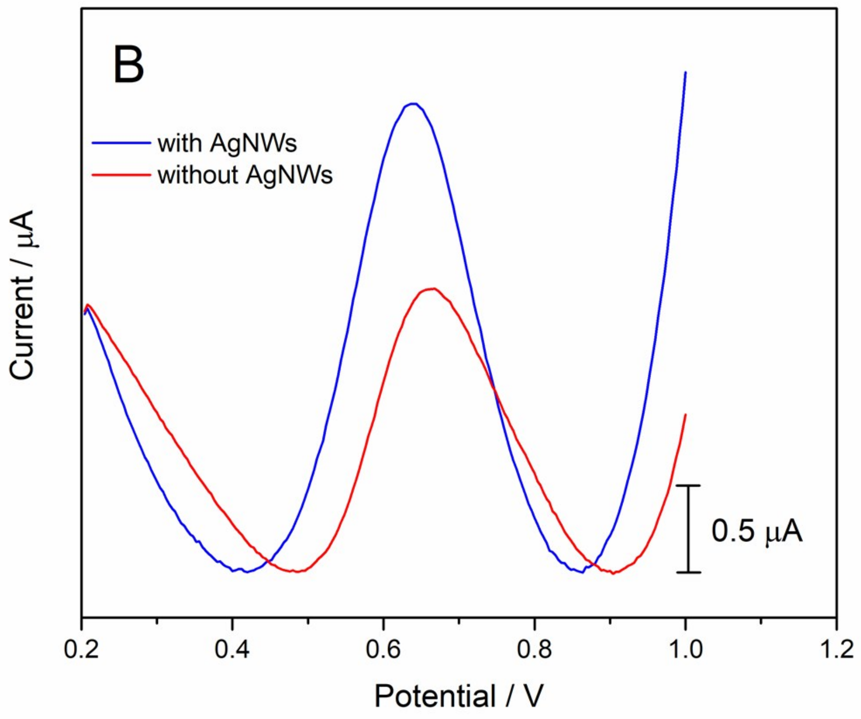
B
with AgNWs
without AgNWs
Current / μA
0.5 μA
Potential / V
0.2
0.4
0.6
0.8
1.0
1.2

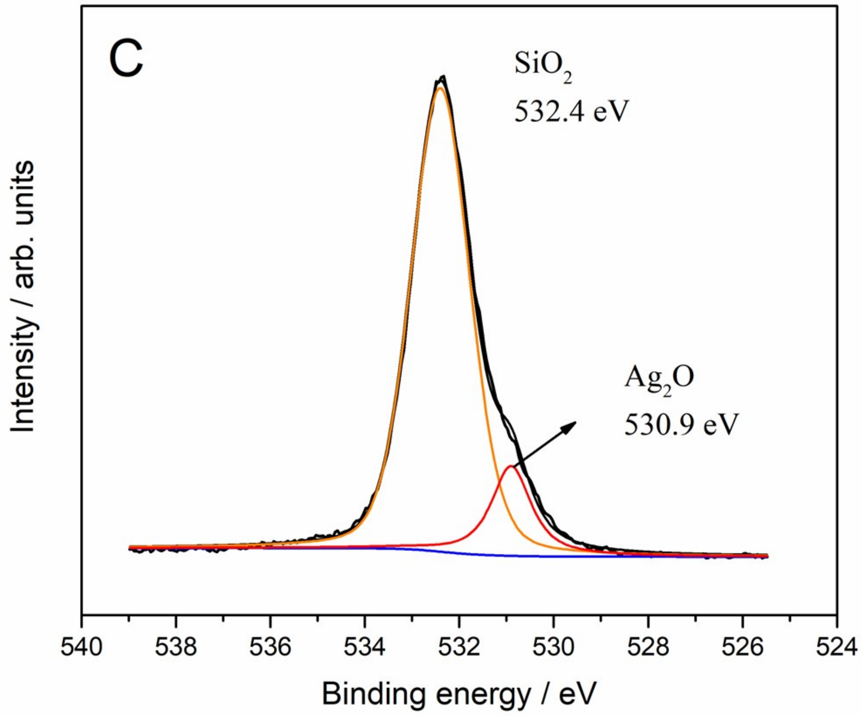

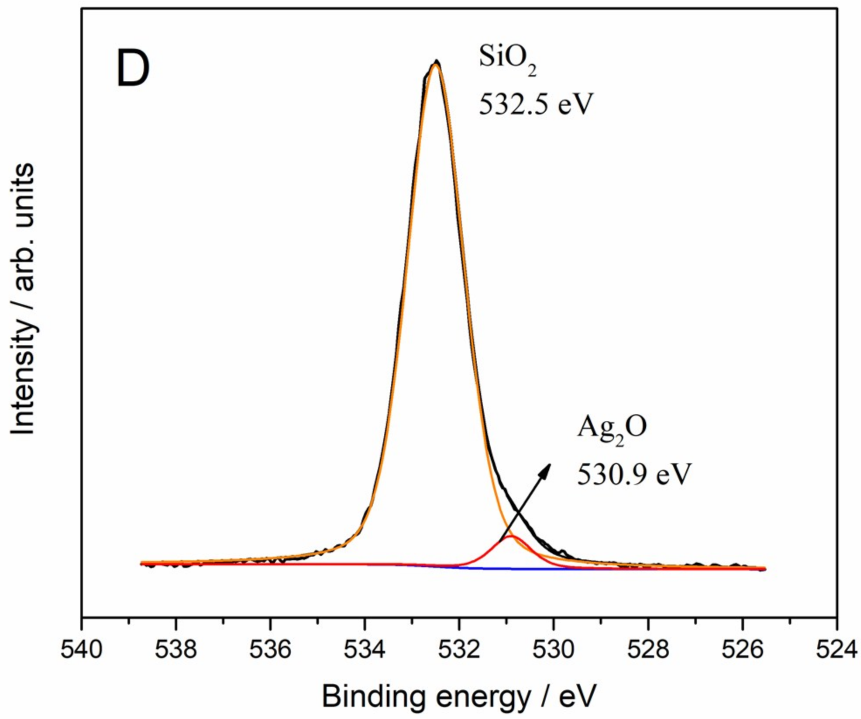

Figure S-1. (A) The CV of (a) GC, (b) AgNWs/GC, (c) Gra/AgNWs/GC, (d) Gra/GC electrodes; (B) The DPV of the AChE/CS/TiO_2-CS/Gra/AgNWs/GC electrode in 1 mM

ATCl; and XPS spectra of O 1s in (C) $AgNWs/SiO_2$, (D) $Gra/AgNWs/SiO_2$ samples after annealed in atmosphere at 120 ℃ for 12 h.

2.2. The effective surface area of electrode

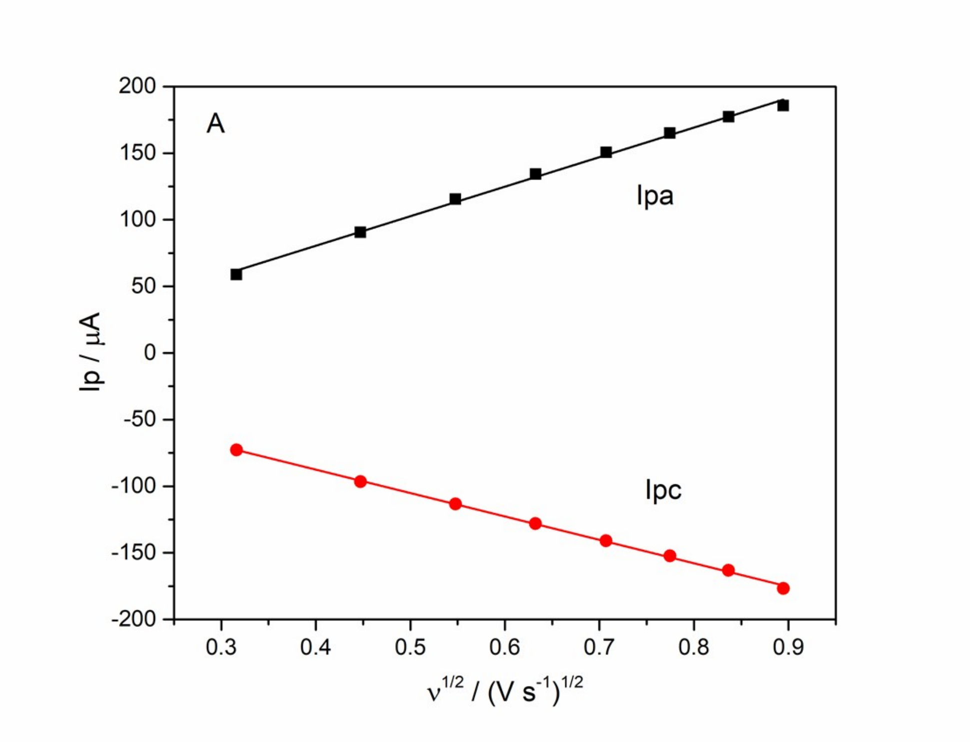

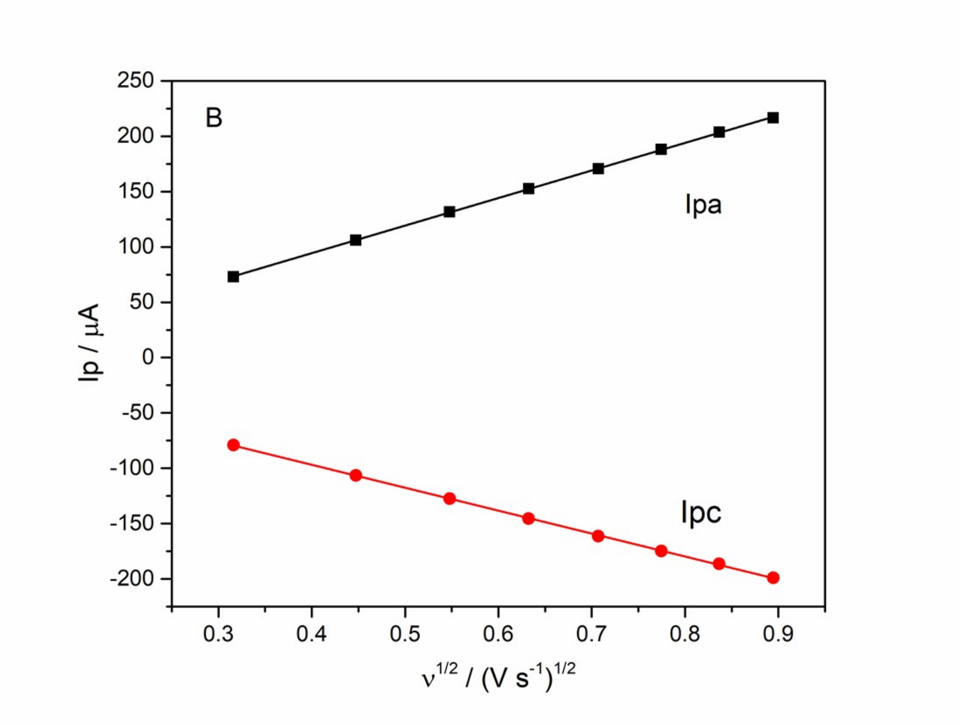

Figure S-2. The anodic peak current (Ipa) and the cathodic peak current (Ipc) vs. the square root of scan rate in the CV curves of the (A) GC electrode, and the Gra/AgNWs/GC electrode in 5 mM $K_3[Fe(CN)_6]$ in 1 M KCl.

2.3. Verify activity of AChE.

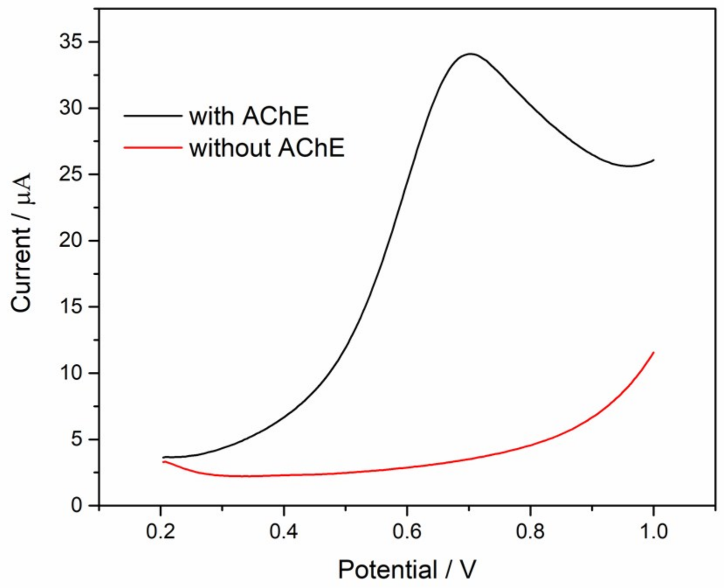

Figure S-3. The DPV responses in 1 mM ATCl of the AChE/CS/TiO_2-CS/Gra/AgNWs/GC (black curve) and CS/TiO_2-CS/Gra/AgNWs/GC (red curve) electrode.

2.4. Storage experiment of the biosensors.

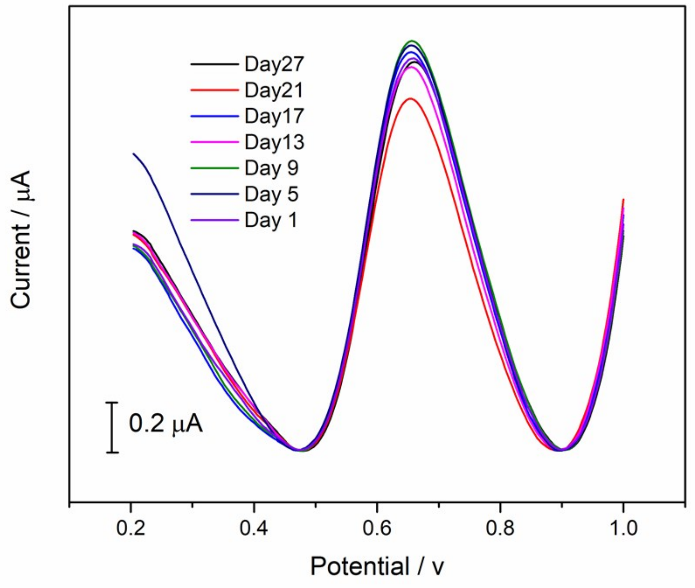

Figure S-4. DPV characterization in 1 mM ATCl of the as fabricated biosensor which was immersed in PBS for 1, 5, 9, 13, 17, 21, 27 days.

2.5. Repetitive experiment of the biosensors.

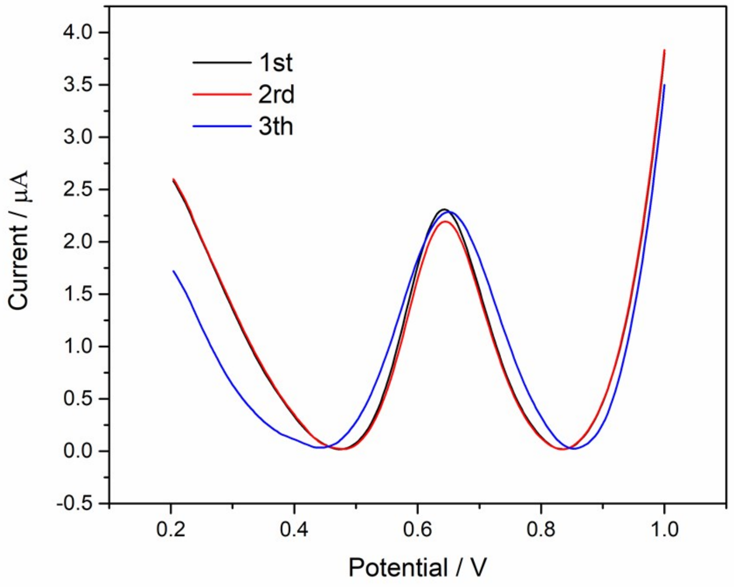

Figure S-5. DPV characterization in 1 mM ATCl of three biosensors prepared continuously.
